# Potential Immune Modularly Role of Glycine in Oral Gingival Inflammation

**DOI:** 10.1155/2013/808367

**Published:** 2013-11-17

**Authors:** Teresa Schaumann, Dominik Kraus, Jochen Winter, Michael Wolf, James Deschner, Andreas Jäger

**Affiliations:** ^1^Department of Orthodontics, Welschnonnenstraße 17, 53111 Bonn, Germany; ^2^Department of Prosthodontics, Preclinical Education, and Material Sciences, Welschnonnenstraße 17, 53111 Bonn, Germany; ^3^Department of Periodontology, Operative and Preventive Dentistry, Welschnonnenstraße 17, 53111 Bon, Germany; ^4^Experimental Dento-Maxillo-Facial Medicine (CRU 208), Welschnonnenstraße 17, 53111 Bonn, Germany

## Abstract

Gingival epithelial cells (GECs) represent a physical barrier against bacteria and are involved in the processes of innate immunity. Recently, an anti-inflammatory and immune-modulatory effect of the amino acid glycine has been demonstrated. However, there is only little information about the immune-modulatory effects of glycine in oral tissues. This study aimed to investigate the existence and role of the glycine receptor in gingival tissue analyzing tissues/cells from extracted human molars via immunohistochemical analysis. *In vitro*, GECs were challenged by inflammatory conditions with IL-1**β** alone or in combination with glycine and analyzed for cytokine expression of IL6/IL8 via real-time PCR. On protein level, the effect of nuclear translocalization of NF**κ**B protein p65 was analyzed using immunofluorescence analysis. A distinct proof of the GlyR in oral gingival tissue and keratinocytes could be demonstrated. Isolated challenge of the keratinocytes with IL-1**β** as well as with glycine resulted in an upregulation of IL6 and IL8 mRNA expression and activation of NF**κ**B pathway. The presence of glycine in combination with the inflammatory stimulus led to a significant decrease in inflammatory parameters. These results indicate a possible anti-inflammatory role of glycine in gingival inflammation and encourage further research on the utility of glycine in the prevention or therapy of inflammatory periodontitis.

## 1. Introduction

Periodontitis typically starts with inflammation of the gingiva and proceeds by spreading into the deeper structures of the periodontium, leading to progressive destruction of periodontal tissues and the alveolar bone and to the loss of teeth [1]. As a major part of the gingival tissue, gingival keratinocytes represent a physical barrier to infections by periodontal pathogens [2]. While the epithelium was previously thought to provide only a passive role in inflammation, recent articles demonstrated a new perspective to inflammatory conditions assigning an active role to the epithelium in the host response to bacterial infections [3]. According to these authors, the epithelium reacts to bacterial challenges by signaling host defense and integrating innate and acquired immune responses. 

Signaling pathways of gingival keratinocytes that are modulated by bacteria such as *Porphyromonas gingivalis* in the course of periodontal infection include changes in intracellular calcium ion (Ca^2+^) concentrations [4, 5]. Izutzu et al. [4] demonstrated that *P. gingivalis* invasion and inflammatory response in human gingival epithelial cells is related to a release of Ca^2+^ from intracellular reservoirs and subsequent increase in cytosolic Ca^2+^. Contact between *P. gingivalis* and epithelial cells is shown to activate the host-cell Ca^2+^ signaling system, a further sign for its inflammatory impact. 

Similar inflammatory signaling is also reported for the cytokine interleukin 1*β* (IL-1*β*) which is involved in the pathogenesis of many inflammatory diseases such as rheumatoid arthritis, inflammatory bowel disease, atherosclerosis, and also periodontitis [6–8]. In human keratinocytes, induction of IL-1*β* was demonstrated to take place upon stimulation with bacterial lipopolysaccharide (LPS), physical or thermal injury, ultraviolet irradiation, and a variety of cytokines, that is, granulocyte-macrophage colony-stimulating factor (GM-CSF), tumor necrosis factor-*α* (TNF-*α*), interleukin 6 (IL6), transforming growth factor-*β* (TGF-*α*), and IL-1*β* [6, 9]. IL-1*β* is reported to trigger chemotaxis of neutrophil granulocytes as well as T and B cell activation. In addition, it stimulates the expression of the early response genes, multiple cytokines, and inflammatory factors that drive extracellular matrix degradation [6, 7]. 

For an anti-inflammatory purpose, the use of glycine is reported to induce beneficial immune-modulatory and cytoprotective effects [10–12]. L-glycine is the smallest nonessential amino acid that consists of a methylene carbon molecule attached to an amino- and a carboxyl group. In nonnervous tissue, glycine is considered to be biologically neutral. In the last years, however, numerous investigations have revealed significant effects of glycine on the activation of cells belonging to the innate as well as the adaptive immune system, including macrophages, polymorphonuclear neutrophils (PMNs), and lymphocytes [13–15]. In patient's treatment, glycine has been reported to have several beneficial effects including protection against toxicity induced by anoxia, oxidative stress, and various toxic agents at the cell, tissue, and whole body levels [10, 12, 16]. For instance, it was demonstrated that a diet enriched with glycine protected against LPS-induced lethality, hypoxia-reperfusion injury after liver transplantation, D-galactosamine-mediated liver injury, and experimental arthritis [10–12, 17].

The glycine receptor (GlyR) is composed of four 48 kDa *α*-subunits and a 58 kDa *β*-subunit and comprises a pentameric complex that forms a chloride-selective trans-membrane channel [18]. In support of pharmacological evidence for the existence of GlyR in nonneuronal cells stated above, recent studies provide molecular evidence for the GlyR in nonneuronal cells. A wide variety of cells such as neutrophils, alveolar macrophages, endothelial cells, and Kupffer cells have been shown to contain glycine-gated chloride channels [10–12, 16]. In addition, Denda et al. [19] demonstrated that the existence of the GlyR in epidermal keratinocytes might play a crucial role in cutaneous barrier homeostasis.

Several reports showed that glycine suppresses formation of inflammatory cytokines [10–12]. As stated above, the production of proinflammatory mediators induced by bacterial LPS depends on the increase in intracellular Ca^2+^, an effect that was demonstrated to be blunted after treatment with glycine. The exact mechanism of how increased intracellular calcium levels are blocked by glycine is not yet completely understood.

In recent investigations, Breivik and coworkers [20] pointed to the anti-inflammatory potential of glycine in oral tissues. In their animal experiments, these authors were able to demonstrate a significant reduction of artificially induced periodontal infection following a specific glycine diet. Substitution of glycine to animal's food prevented animals from severe periodontal breakdown. However, information is missing whether glycine and its receptors may be able to modulate the development of inflammatory oral gingivitis and if they are able to protect oral tissues from severe periodontal disease.

In this context, the first aim of the present study was expression analysis of glycine receptors in gingival tissue and further analysis of the glycine signaling role in oral gingival keratinocytes. We hypothesized that glycine receptors are also expressed within the oral gingival tissue and that glycine application might be able to modulate the inflammatory response of gingival keratinocytes in inflammatory conditions.

## 2. Material and Methods

All experimental protocols were reviewed and approved by the Ethics Committee of the University of Bonn.

### 2.1. Human Gingival Tissue

Tissue samples from human gingiva were obtained during routine extraction treatment of third molars. Human gingiva samples were collected from three different human donors aged between 12 and 14 years showing no clinical signs of gingivitis and periodontitis. The teeth had been extracted for orthodontic reasons and with written parental consent. Following extraction, human teeth including gingiva were perfused with phosphate-buffered saline supplemented with 4% paraformaldehyde for fixation purposes. Afterwards, the gingiva of each tooth was dissected and prepared for light microscopical examination as recently described [21, 22].

### 2.2. Histology

For histological analyses, all specimens were processed for paraffin histology. To perform histology, 5–7 *μ*m serial sagittal sections of each specimen were prepared. For orientation purposes, selected sections were stained with hematoxylin and eosin.

### 2.3. Immunohistochemistry

To analyze glycine receptor expression, tissue sections were processed for immunohistochemical detection of glycine receptor expression according to previously established protocols [22, 23]. In brief, sections were incubated with primary antibody of rabbit origin raised against a peptide mapping at the carboxy terminus of the protein (anti-glycine receptor, SYSY, Göttingen, Germany) in a 1 : 250 working solution of TBS/BSA at 4°C overnight in a humidified chamber. A 1 : 100 dilution of a goat anti-rabbit immunoglobulin (Dako A/S, Denmark) was incubated as secondary antibody for 30 min. Following further rinsing, a PAP complex (1 : 150 in TBS/BSA; Dako A/S Denmark) was administered for 30 min prior to the visualization of antibody binding with 3,3′-diaminobenzidine (Sigma Chemicals, USA) solution for about 5 min. Thereafter, specimens were counterstained with Mayer's hematoxylin, dehydrated, and cover-slipped for light microscopical analysis.

In order to prove the specificity of the immunoreactions, negative controls were carried out by (a) omitting the primary antibody or using nonimmune IgG instead and (b) omitting both the primary and secondary antibody and using TBS/BSA instead.

Preadsorption controls were used to exclude unspecific binding of the antibodies to unrelated antigens. The antibody was combined with a twofold excess of blocking peptide and incubated at 4°C overnight. Following neutralization, the antibody/peptide mixture was diluted into the appropriate working solution. Afterwards, immunohistochemistry was carried out as described above.

### 2.4. Explantation of Gingival Keratinocytes

Gingival keratinocytes/epithelial cells (GECs) were cultured from gingiva of three periodontal healthy patients as described above. The cells were isolated by collagenase digestion and subsequent mechanical isolation of the epithelial layer from the connective tissue as described previously [24]. Following explantation, cells were seeded on 60 mm dishes and cultured in KGM-Medium (Keratinocyte Growth Medium, PromoCell, Heidelberg, Germany) supplemented with CaCl_2_ (0.15 mM) and 0.5% antibiotics (diluted from a stock solution containing 5,000 U/mL penicillin and 5,000 U/mL streptomycin; Biochrom AG, Germany) under a saturated humidified atmosphere containing 5% CO_2_ at 37°C. The medium was changed every other day. Prior to experimental use, gingiva keratinocytes were characterized as described previously [24]. For cell culture experiments, cells were used in passage 4.

### 2.5. Induction of Gingival Inflammation: *In Vitro*

To mimic inflammatory impact on gingival keratinocytes similar to the environment in gingivitis, cells were cultured in presence of the amount of 5 ng/mL IL-1*β* (Promokine, Heidelberg, Germany) as measured in patients suffering from gingival infection and proven to be effective to induce inflammatory response [24–26]. For experiments *in vitro*, cells were grown to a confluent state (90%). One hour prior to stimulation, cells were adapted to a glycine-free medium (SMEM, Supplemented Eagle's Minimum Essential Medium, GIBCO/Invitrogen, Darmstadt, Germany). For stimulation experiments, cells were treated with 5 ng/mL IL-1*β* in the presence or absence of glycine (5 mM glycine). Following a stimulation time of 30 or 60 minutes, cells were analyzed for changes in gene expression of proinflammatory markers IL6 and IL8. Preliminary experiments demonstrated a 30 min stimulation time for IL6 and 60 min stimulation time for IL8 to be most effective for inflammatory response induction.

### 2.6. Glycine Stimulation Experiments

To analyze the possible anti-inflammatory effect of glycine on gingiva keratinocytes, in addition to the IL-1*β* conditioned medium, cells were additionally stimulated with 5 mM glycine (Sigma-Aldrich, Taufkirchen, Germany) as demonstrated to be effective previously by Vardar-Sengul et al. [26]. For negative control, glycine was administered alone.

### 2.7. Analyses of NF*κ*B Pathway Activation

To further characterize pro- or anti-inflammatory effects of IL-1*β* with or without glycine, activation of NF*κ*B pathway was investigated. According to a protocol published previously [24], gingival keratinocytes were stimulated for 6 h in combination with IL-1*β* with and without glycine as described above. After incubation, cells were fixed and further analyzed for nuclear translocation of p65 immune reactions using immune cytochemistry with a primary antibody against NF*κ*B (BioLegend, Uithoorn, Netherlands) in a 1 : 100 concentration [7]. The amount of cells showing positive immunoreactions over the cell nucleus was assessed semiquantitatively by a modification of the grading system published previously [21]. The grading system was set up as follows: 0 = no immune reaction in cell nucleus; 1 = 1/3 of the cell nucleus demonstrated immune reactions; 2 = 2/3 of the cell nucleus demonstrated immune reactions; 3 = immune reaction all over the cell nucleus and the cytoplasm were visible; 4 = immune reactivity was only located in cell nucleus. Cells were counted in relation to total cell number at the investigated region using AxioVision software (Carl Zeiss, Germany). To ensure reproducibility, all counts were performed on four independent experiments per stimulation group.

### 2.8. RNA Extraction and cDNA Synthesis

RNA was isolated using the “RNeasy Protect Mini Kit” (Qiagen, Hilden, Germany) and quantified using the NanoDrop ND-1000 Spectrophotometer (NanoDrop, Technologies, Wilmington, USA). 1 *μ*g total RNA was reversely transcribed using “iScript Select cDNA Synthesis Kit” (BioRad, Munich, Germany) with oligo(dT)-primers [24].

### 2.9. Quantitative Real-Time PCR

Gene expression of *β*-actin, IL6, and IL8 was analyzed by real-time PCR with the iCycler Thermal Cycler (BioRad). SYBR Green served as fluorophore for online monitoring of generated PCR products as described previously [24]. In brief, all primers were synthesized according to the highest quality standards (Metabion, Martinsried, Germany) and verified by computer analysis for specification (BLAST). Primer sequences are presented in Table 1. Amplification for the detection of *β*-actin was performed under the following conditions: 95°C for 5 min followed by 40 cycles of 95°C for 15 s, 69°C for 30 s, and 72°C for 30 s. IL6 and IL8 were amplified under the same conditions except for the annealing temperature which was set at 68°C. 50 ng cDNA was added to a master mix containing primers and IQ SYBR Green Supermix (BioRad). The reference gene *β*-actin was used as standard for normalizing the crossing point. Cloned PCR products derived from the specific primers served as positive controls for the PCR, while water was used as the negative control. PCR was performed for all samples individually. Resulting gene expressions were averaged. Relative differential gene expression was calculated using the method described by Pfaffl [27]. PCR efficiencies were determined with dilution series as listed above (Table 2).

### 2.10. Immunofluorescence and Cytochemistry

GECs were cultured on sterile coverslips and grown to 90% of confluency. The cells were washed with phosphate-buffered saline (PBS, PAA Laboratories, Cölbe, Germany), fixed with 4% paraformaldehyde for 15 min at room temperature, washed with PBS, and permeabilized thereafter in 0.1% Triton X-100 in PBS for 10 min. After washing with PBS, nonspecific binding was blocked by a 60 min treatment in a 1% bovine serum albumin solution (BSA; Sigma-Aldrich). Incubation with the primary anti-glycine receptor antibody (SYSY, Göttingen, Germany, 1:250) was performed overnight at room temperature. Following extensive washing, a Cy-3-conjugated anti-rabbit IgG secondary antibody (Dianova, Hamburg, Germany) was applied for 50 min at room temperature. Finally, cells were washed three times with PBS. Nuclear staining was achieved by incubating the cells in DAPI for 8 min, followed by washing twice with Aqua dest. Cells were mounted with Mowiol/DABCO (Roth, Karlsruhe, Germany) for fluorescence microscopic imaging using a Zeiss fluorescence microscope (AXIO Imager A1, Carl Zeiss MicroImaging). For cytochemistry, a horseradish-peroxidase- (HRP-) conjugated secondary antibody (Dako Invision) was used instead and cells were counterstained with hemalaun as described above.

### 2.11. Statistical Analysis

The PCR data are presented as means ± SD of 10 independent experiments. Statistical significant differences in means were assessed using one-way analysis of variances (ANOVA) in GraphPad Prism version 4.03 for Windows (Graph Pad Software, San Diego, California, USA, http://www.graphpad.com/). A value of *P* < 0.05 was considered statistically significant.

## 3. Results

### 3.1. Presence of the Glycine Receptor in Human Gingival Tissue

**Figure 1:**
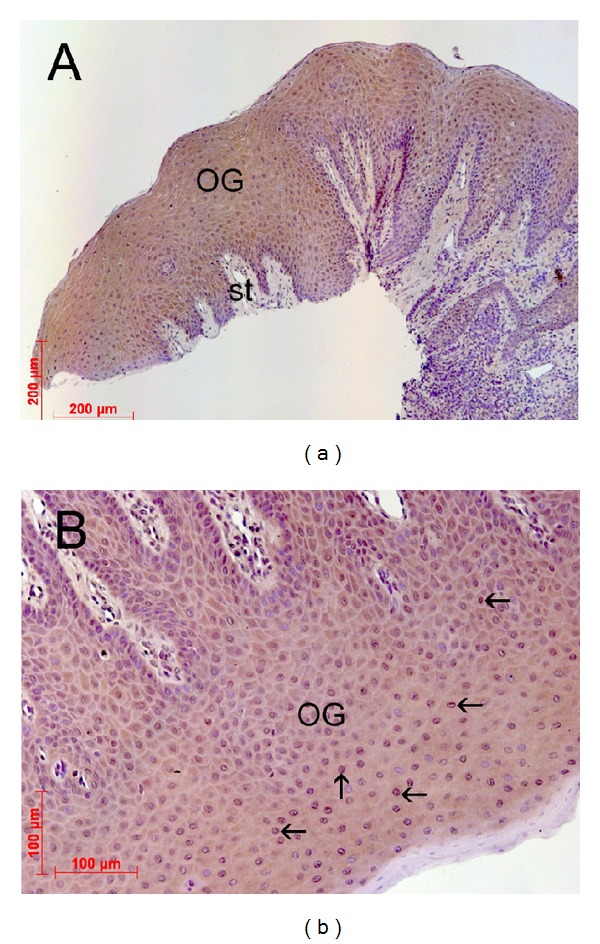
Demonstration of the glycine receptor (GlyR) in gingival tissue. Overview (a) of oral tissue (magnification ×10) isolated from extracted upper third molars showing different region of the gingival tissue (OG: oral gingival tissue; st: subepithelial tissue). (b) Representative gingival tissue section (magnification ×200) assessed by immunohistochemistry using an anti-glycine receptor antibody (SYSY, Göttingen, Germany) at 4°C overnight and counterstaining with DAB (brown color). There was strong immune reaction in the epithelial layers of the gingiva, namely, in gingival keratinocytes. The underlying basal membrane as well as the connective tissue showed negative immune staining.

Explants of human gingival tissue were analyzed for glycine receptor expression by immunohistochemistry to investigate whether glycine receptor is expressed in human gingival tissue. The immune staining of healthy human gingival tissue sections revealed a strong glycine receptor expression within the epithelial layers of the gingiva tissue. Within the investigated samples, glycine receptor immune reactivity was mainly concentrated to gingival keratinocytes (Figures 1(a) and 1(b)). At cellular level, positive immune reactions were mainly located at both cell membranes and in the cytoplasm of the cells. The underlying basal membrane of the gingiva tissue as well as the other connective tissues showed no positive immune reactions for the glycine receptor.

### 3.2. Demonstration of Glycine Receptor in Isolated Gingiva Keratinocytes

**Figure 2:**
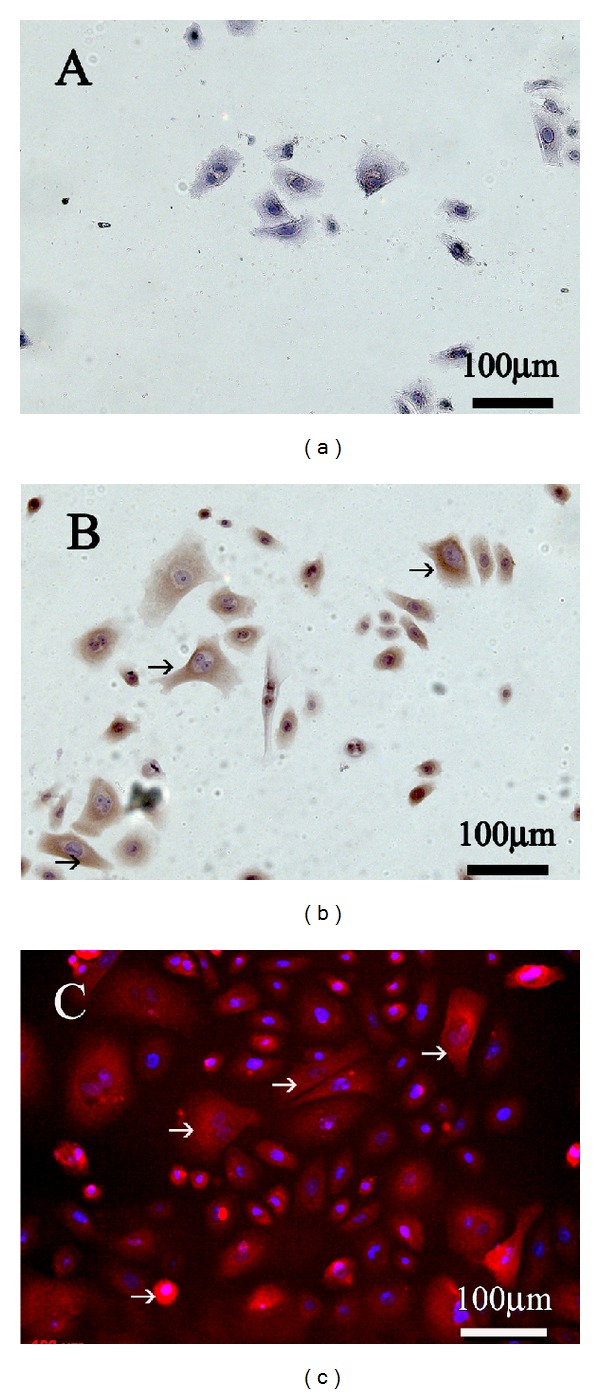
Cytochemical ((a): negative control, (b): anti-glycine receptor) and immunofluorescence ((c) anti-glycine receptor) staining for Glycine receptor in cultured human gingival keratinocytes (red color, black arrow). For nuclear staining, cells were treated with DAPI (blue color, (c)).

In a second step, the aim was to verify the observed *in vivo* findings of glycine receptor expression in human gingival tissue sections in isolated human gingival keratinocytes in order to provide the basis for further experiments on those cells. Similar to the *in vivo* findings, positive immune reactions for glycine receptor were also found in cell cultures of isolated gingival keratinocytes. In our experiments fourth passage gingival keratinocytes from healthy humane donors demonstrated a significant amount of immune reactions for glycine receptor expression mainly located on the outer cell membrane and in the cell cytoplasm (Figures 2(a), 2(b), and 2(c)).

### 3.3. Application of Isolated Challenge with IL-1*β*

**Figure 3:**
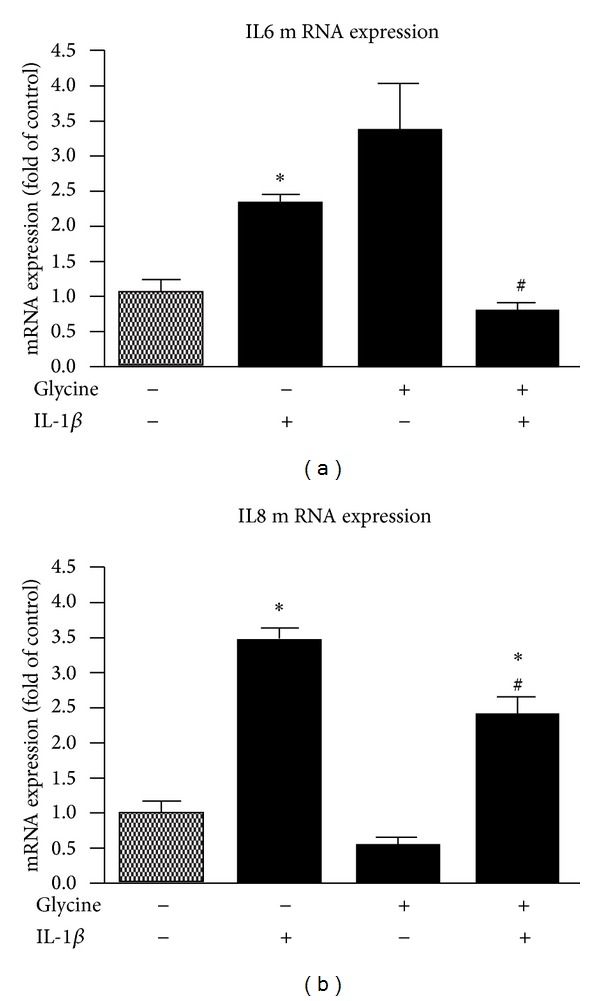
Effect of IL-1*β* and glycine treatment on the mRNA expression of the proinflammatory cytokines IL6 (a) and IL8 (b). Confluent cultures of gingival keratinocytes were treated with IL-1*β* either alone or in combination with glycine to induce inflammatory conditions *in vitro*. Glycine administration to inflammatory challenged gingival keratinocytes resulted in a decreased proinflammatory cytokine mRNA expression of both IL6 (a) and IL8 (b). Results are presented as means ± SD (*n* = 10); *P < 0.05 difference between groups following ANOVA analysis.

To mimic an inflammatory impact on gingival keratinocytes similar to conditions in oral gingivitis in patients, cells were cultured in presence of the amount of IL-1*β* which was measured in gingival inflamed patients previously [26]. Following IL-1*β* stimulation, an impact on mRNA expression of proinflammatory markers interleukin 6 and interleukin 8 was observed. Compared to untreated controls, the mRNA expression of IL6 was increased by 2.3-fold (Figure 3(a)) and IL8 expression was upregulated by 3.5-fold (Figure 3(b)). Changes in IL6 and IL8 were demonstrated to be significant to control cultures.

### 3.4. Effect of Glycine on IL6 and IL8 mRNA Expression

In addition to the IL-1*β* administration, cells were additionally stimulated with glycine to analyze the anti-inflammatory potential of glycine on induced gingival inflammatory conditions. Following glycine administration, the expression of the analyzed proinflammatory markers was demonstrated to be downregulated. The observed inflammatory effect on IL6 expression was significantly reduced compared to the isolated IL-1*β* stimulation group (0.8- instead of 2.3-fold of control; Figure 3(a)). The effect on IL8 mRNA expression was also significantly reduced when glycine was added to the conditioned cell culture medium (2.4- instead of 3.5-fold of control (Figure 3(b)). Interestingly, isolated application of glycine seems also to induce a slight upregulation of IL6 in mRNA expression (Figure 3(a)). In combination with IL-1*β* a downregulation was observed.

### 3.5. Effect of Glycine on NF*κ*B Pathway-Mediated Inflammatory Response

**Figure 4:**
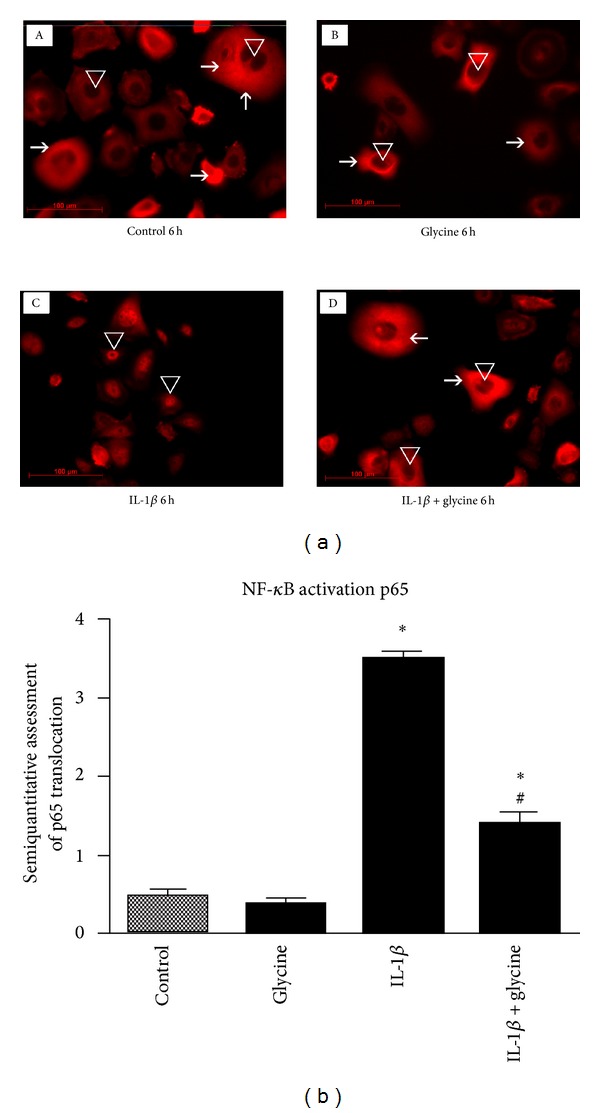
Effects of IL-1*β* and/or glycine on NF*κ*B signaling. (a) Immune fluorescence staining of NF*κ*B signaling protein p65 of cells (A), glycine (B), IL-1*β* (C), and IL-1*β* + glycine (D) treated human gingival keratinocytes. The photographs demonstrate a localization of p65 protein primarily in the cell cytoplasm in untreated cells. Untreated controls (A) and glycine treated cells (B) did not affect p65 expression. Following the induction of inflammation by IL-1*β* administration, NF*κ*B pathway was activated as demonstrated by translocation of p65 protein to the cell nucleus (C). The addition of glycine to the inflammatory cell culture medium reduced the observed p65 protein translocation (D). (b) Semiquantitative assessment of p65 protein translocation in human gingival keratinocytes following administration of IL-1*β* in combination with and without glycine. *P < 0.05 difference between treated and control groups following ANOVA analysis; ^#^ *P* < 0.05 difference between IL-1*β* and IL-1*β* + glycine treated groups.

As a second approach, the immune-modulatory effect of glycine on inflammatory signaling pathway NF*κ*B using the NF*κ*B related protein p65 was analyzed. In this context, changes were addressed for p65 protein expression which is localized in the cytoplasm in noninflammatory conditions. In the present experiments, p65 protein was demonstrated to be located in the cell cytoplasm of untreated human gingival keratinocytes (Figure 4). Following the induction of inflammation by stimulation with IL-1*β*, a pronounced accumulation of NF*κ*B in the cell nucleus was observed. When glycine was added to the conditioned cell culture medium the inflammatory translocation of p65 NF*κ*B pathway protein was almost completely abolished. Similar to control experiments, p65 immune reactions were mainly located in the cytoplasm of the cells and did not translocate to the cell nucleus in glycine treated cell culture experiments.

## 4. Discussion

To our knowledge, this study is the first which analyzes the existence of the glycine receptor in gingival tissue and gingival derived keratinocytes. Both, tissue sections of human gingival tissue and human gingival keratinocytes demonstrated a ubiquitous expression of the glycine receptor in the gingival tissue which was mainly located at the cell membrane and in the cytoplasmic region. Similar to present findings, other authors documented similar findings on the location of the glycine binding receptor (GlyR) in different cell systems. For example, Webb and Lynch demonstrated in their studies on neural cells that the glycine receptor was located on the cell membrane which acts as a receptor with transmembrane domains and extracellular binding sites as well as chloride-sensitive transmembrane channels [28]. Similar to this report, Zhong et al. [11] also demonstrated the GlyR as a membrane receptor in “Kupffer” cells. On the other hand, Béchade [29] observed both a membrane and a more cytoplasmatic localization of the GlyR in oligodendrocytes and in glia cells. From the present findings the expression of GlyR both in the membrane and within the cell cytoplasm in gingival keratinocytes can be suggested.

To mimic the clinical situation of gingival inflammation such as in conditions of oral gingival inflammation in the present *in vitro* experiments, isolated gingival keratinocytes were challenged with IL-1*β* at the amount being found to be expressed in patients suffering from gingivitis [30]. The used amount of interleukin 1*β* application has been proven to be effective in the present findings as shown by the upregulation of cytokine mRNA expression levels of IL6 and IL8 and the induction of NF*κ*B activation in treated gingival keratinocytes. Steinberg et al. [7] and McKay and Cidlowski [31] also reported similar effects of IL-1*β*-induced inflammation in their experiments on gingival keratinocytes after stimulation with IL-1*β*. Also the inflammatory response of oral cells to cytokine application on protein level of the investigated inflammatory parameters was proven in previous experiments suggesting the effectiveness of the present protocol [24, 32, 33]. A significant inflammatory response of treated specimens was observed in this study and also in reports from other authors. Corresponding to the presented regulation of IL6 and IL8 mRNA expression, also Kraus and coworkers [24] presented in a recent work that bacterial LPS stimulation was able to show similar and even stronger inflammatory responses of gingival keratinocytes as documented in the present study. Again the present data on IL-1*β* induced inflammation underline the efficiency and capability of IL-*β* to induce inflammatory reactions. 

The addition of glycine to the cell culture medium of inflammatory challenged gingival keratinocytes demonstrated the ability to attenuate the observed inflammatory response of gingival keratinocytes. Since the standard Keratinocyte Growth Medium (KGM) contains different amino acids including glycine, we therefore analyzed its impact on cell culture conditions by using also glycine-free medium in preliminary unpublished experiments. Induction of different inflammatory cytokines in keratinocytes was demonstrated to be nearly identical compared to the used standard medium in these experiments. Therefore the described standard cell culture medium was used in present experiments. 

Interestingly an initial upregulation of the mRNA expression of the investigated early inflammatory marker IL6, but not IL8, after 30 min of stimulation with glycine alone was observed. A possible explanation for this regulation might be due to an early cellular response to changes within the cell culture medium by the application of glycine. Interestingly in combination with IL-1*β* protein this upregulation was inhibited. The regulation of IL8 expression, which is known as a more stable inflammatory marker, was not affected by glycine application. After adding glycine to the cytokine contaminated medium, a significant inhibition of the immediate upregulation of the cytokines IL6 and IL8 at the transcriptional level and a decrease in p65 nuclear translocation were observed. This glycine-induced anti-inflammatory response goes hand in hand with reports of others on observations in several different cell and organ systems. In his recent review article Zhong and coworkers [11] pointed to the observed anti-inflammatory potential of glycine. They reported that glycine is found to be protective under several inflammatory conditions such as shock, endotoxin, and sepsis and to prevent ischemia-reperfusion injury to a variety of tissues and organs including liver, kidney, heart, intestine, and skeletal muscle. Furthermore, glycine also protected against peptidoglycan polysaccharide-induced arthritis and protects the gastric mucosa against chemical and stress-induced ulcers. In their review these authors concluded that glycine appears to exert several protective effects, including anti-inflammatory, immune-modulatory, and direct cytoprotective actions [11]. Furthermore it has been suggested that glycine is also able to act on inflammatory cells such as macrophages to suppress activation of inflammatory transcription factors and the formation of free radicals and inflammatory cytokines. In the light of the findings in the present investigation and the reported effects of glycine in the literature, it can be suggested that also gingival tissue with special regard to gingival keratinocytes is able to perform a glycine-mediated anti-inflammatory response under inflammatory circumstances.

## 5. Conclusions

In conclusion, the present data demonstrate at first that the glycine receptor is expressed in gingival tissue and indicates an immune-regulatory role for glycine in the response of gingival keratinocytes to inflammatory conditions. Together with recent reports about glycine physiology, the modulatory potential of glycine within the inflammatory process in gingival tissue becomes obvious. The mapping of intercellular cytokine signaling networks and its modulating reagents, with special regard to glycine that functionally couples gingival tissues, and its activation to induce immune response may also provide information for future potential clinical targets. This could include the prevention of severe gingivitis and the further development of periodontal disease which is discussed to be the result of long lasting gingival inflammation. Furthermore, the immune-modulatory role of glycine in the course of gingival inflammation extends the well accepted knowledge of anti-inflammatory effects of glycine in the oral gingival tissue that again points to glycine as a promising treatment agent in inflammatory conditions.

## Figures and Tables

**Table 1:** Primer sequences for real-time PCR for *β*-actin, IL-6, and IL-8.

Gene	Primer sequence
*β*-actin	Sense 5′-CATGGATGATGATATCGCCGCG-3′
	Antisense 5′-ACATGATCTGGGTCATCTTCTCG-3′
IL-6	Sense 5′-ATGAACTCCTTCTCCACAAGC-3′
	Antisense 5′-CTACATTTGCCGAAGAGCCC-3′
IL-8	Sense 5′-ATGACTTCCAAGCTGGCCGTGG-3′
	Antisense 5′-TGAATTCTCAGCCCTCTTCAAAAAC-3′

**Table 2:** Primer efficiencies and corresponding annealing temperatures for *β*-actin, IL-6, and IL-8.

Gene	Efficiency	Temperature (°C)
*β*-actin	1.84	69
IL-6	2.12	68
IL-8	2.02	68
